# Gcn4p and Novel Upstream Activating Sequences Regulate Targets of the Unfolded Protein Response

**DOI:** 10.1371/journal.pbio.0020246

**Published:** 2004-08-17

**Authors:** Christopher K Patil, Hao Li, Peter Walter

**Affiliations:** **1**Howard Hughes Medical Institute, Chevy ChaseMaryland, United States of America; **2**Department of Biochemistry and Biophysics, University of CaliforniaSan Francisco, California, United States of America; **3**California Institute for Quantitative Biomedical Research, San FranciscoCaliforniaUnited States of America

## Abstract

Eukaryotic cells respond to accumulation of unfolded proteins in the endoplasmic reticulum (ER) by activating the unfolded protein response (UPR), a signal transduction pathway that communicates between the ER and the nucleus. In yeast, a large set of UPR target genes has been experimentally determined, but the previously characterized unfolded protein response element (UPRE), an upstream activating sequence (UAS) found in the promoter of the UPR target gene *KAR2,* cannot account for the transcriptional regulation of most genes in this set. To address this puzzle, we analyzed the promoters of UPR target genes computationally, identifying as candidate UASs short sequences that are statistically overrepresented. We tested the most promising of these candidate UASs for biological activity, and identified two novel UPREs, which are necessary and sufficient for UPR activation of promoters. A genetic screen for activators of the novel motifs revealed that the transcription factor Gcn4p plays an essential and previously unrecognized role in the UPR: Gcn4p and its activator Gcn2p are required for induction of a majority of UPR target genes during ER stress. Both Hac1p and Gcn4p bind target gene promoters to stimulate transcriptional induction. Regulation of Gcn4p levels in response to changing physiological conditions may function as an additional means to modulate the UPR. The discovery of a role for Gcn4p in the yeast UPR reveals an additional level of complexity and demonstrates a surprising conservation of the signaling circuit between yeast and metazoan cells.

## Abstract 2

The yeast unfolded protein response activates a large set of target genes, but a characterized element found in the promoter of one target, KAR2, cannot account for most targets. Using computational and experimental methods, the authors identify additional elements, as well a role for GCN4p in the response

## Introduction

The vast majority of all cellular secretory and membrane proteins are folded and modified in the endoplasmic reticulum (ER), from which they are transported to their final destination in the secretory pathway. When the protein folding capacity of the ER is exceeded or experimentally impaired, unfolded proteins accumulate in the ER and activate the unfolded protein response (UPR). The UPR allows the ER to communicate with the nucleus (Patil and Walter 2001), where a comprehensive gene expression program is induced to adjust the protein folding capacity of the cell according to need.

In the yeast *S. cerevisiae,* unfolded ER proteins stimulate the ER-resident bifunctional transmembrane kinase/endoribonuclease Ire1p (Cox et al. 1993; Mori et al. 1993; Sidrauski and Walter 1997). When activated, Ire1p excises a 252-nucleotide intron from the mRNA encoding Hac1p, a bZIP transcription factor required for induction of all UPR target genes (Cox and Walter 1996; Mori et al. 1996; Sidrauski and Walter 1997). Removal of the *HAC1* intron and subsequent ligation of the two liberated exons by tRNA ligase (Sidrauski et al. 1996) produces a spliced mRNA that is efficiently translated (Kawahara et al. 1997). In the absence of splicing, the intron blocks translation of the mRNA (Rüegsegger et al. 2001). Splicing is therefore a prerequisite for Hac1p production and thus serves as the key regulatory step in the UPR. When it is produced, Hac1p binds an upstream activating sequence (UAS), the unfolded protein response element (UPRE), found in the promoters of UPR target genes (Mori et al. 1992; Kohno et al. 1993), thereby stimulating the transcriptional response to protein unfolding.

Several salient features of the UPR are conserved between yeast and metazoans. In metazoans, Ire1p orthologs Ire1-α and Ire1-β remove a short intron from the *XBP-1* mRNA, which encodes a bZIP transcription factor analogous to Hac1p (Wang et al. 1998; Miyoshi et al. 2000; Urano et al. 2000; Calfon et al. 2002). The metazoan UPR, however, is implemented by at least two additional ER-resident sensors, which are thought to act in parallel and induce multiple downstream transcriptional activators not known to exist in yeast. A second branch of ER-to-nucleus signaling is mediated by ATF-6, a bZIP transcription factor that is synthesized as an integral ER transmembrane protein (Haze et al. 1999). Upon UPR induction, ATF-6 is proteolytically cleaved, liberating a soluble fragment that moves to the nucleus to induce transcription in association with XBP-1 (Wang et al. 2000; Ye et al. 2000; Steiner et al. 2001; Yoshida et al. 2001; Lee et al. 2002a). A third branch of the metazoan UPR provides translational control by the ER transmembrane kinase PERK (Harding et al. 1999; Liu et al. 2000). When activated in response to protein misfolding in the ER, PERK phosphorylates the translation initiation factor eIF-2α, thereby down-tuning translation of many mRNAs (and decreasing the translocational load on the ER) (Harding et al. 2000a, 2000b). Under conditions of limiting eIF-2α activity, however, some mRNAs containing short upstream open reading frames (ORFs) in their 5′ UTR are preferentially translated. One of these mRNAs encodes a third bZIP transcription factor, ATF-4, which collaborates with XBP-1 and other cellular stress signaling factors to activate UPR targets (Harding et al. 2000a, 2003; Ma et al. 2002).

The UPR target genes of yeast have been comprehensively defined by microarray expression profiling, where they comprise a significant fraction of the yeast genome (381 genes, more than 5% of the ORFs) (Travers et al. 2000). The UPR target genes encode many proteins that play critical roles in the ER, the Golgi apparatus, and throughout the secretory pathway. Hence, the UPR can be thought of as a means of homeostatic control, serving to remodel the secretory pathway according to the cell's need.

The set of 381 genes was defined by microarray hybridization expression profiling, using a stringent quantitative “filter” that required the expression profile of each target gene to closely match that of previously known and well-characterized UPR target genes. In particular, the filter demanded that the expression profile of a target gene closely correlate to that of canonical UPR targets over a time course of UPR induction, and that induction be significantly greater in wild-type (WT) than in either Δ*ire1* or Δ*hac1* cells.

The identification of this vast set of target genes poses an enigma in light of the previously characterized UPRE. The UPRE was originally defined as a 22-bp sequence element of the *KAR2*/BiP promoter (Mori et al. 1992) and subsequently carefully refined to nucleotide precision as a semipalindromic seven-nucleotide consensus, CAGNGTG (Mori et al. 1998). Point mutations in any one of the six conserved nucleotides or deletion of the central nucleotide was shown to have severely detrimental effects on the ability of the element to function as an autonomous UAS when placed into an otherwise silent promoter. Yet, inspection of the 381 promoter sequences of the experimentally defined set of target genes failed to reveal a recognizable UPRE in most of them. This observation is particularly surprising given that the UPRE is thought to be the Hac1p binding site, and *HAC1* has been shown to be required for activation of all UPR target genes. One possible resolution to this paradox is that additional, heretofore unrecognized UPREs exist that are required for the activation of the genes lacking the “classical” UPRE. A requirement for new *cis*-activating sequences in the promoters of UPR target genes raises the possibility that such sequences could be bound by other *trans*-acting factors, alone or in combination with Hac1p, and thus contribute to the transcriptional complexity of the UPR.

## Results

### Computational Identification of Target Motifs

To identify sequence motifs shared by the set of UPR target genes, we employed a bioinformatics approach to build a “dictionary” of putative regulatory elements from the promoters of these genes. In this approach, DNA sequence is considered as a “text” (a long string of nucleotides), which is modeled as having been composed by concatenating “words” (short oligonucleotides) drawn from a probabilistic “dictionary” according to their frequencies. To infer the dictionary from the observed text, we employed the previously developed computational algorithm, MobyDick, which was developed based on a probabilistic segmentation model (Bussemaker et al. 2000a, 2000b). MobyDick has been used previously to identify regulatory sites in large sets of promoters activated during sporulation or by specific cell-cycle stage.

We first constructed a dictionary from the UPR target gene promoters. To this end, we compiled a text from the promoters of all 381 UPR target genes as previously defined (Travers et al. 2000). We defined the promoter region for each ORF as the 600 nucleotides upstream of the initiation codon. Probabilistic segmentation analysis using the MobyDick algorithm indicated that the target gene promoters are best modeled by a dictionary of about 300 words of eight nucleotides or less (for details of this and subsequent calculations, see Materials and Methods; a complete report of the dictionary with associated statistics appears in Table S1). These words represent the sequences that are most frequent in the target gene promoters.

**Figure 1:**
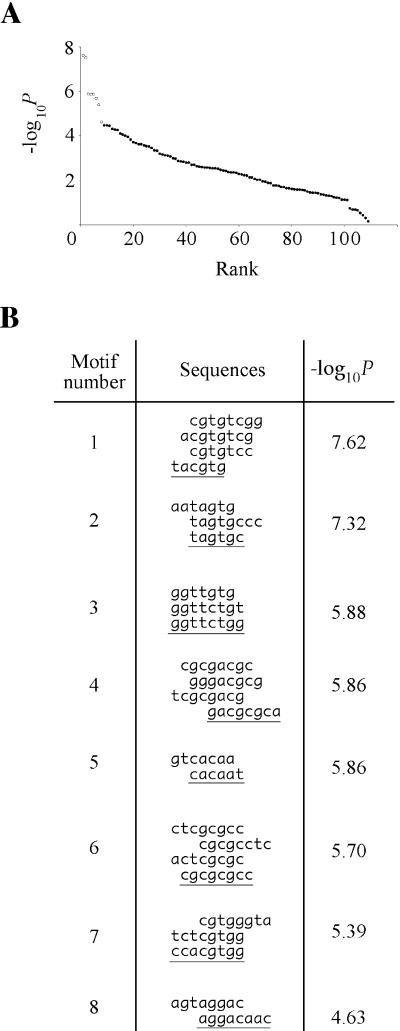
Computational Selection of Candidate Regulatory Motifs (A) Candidate regulatory motifs are overrepresented in UPR target promoters. Sequence motifs were ranked in order of overrepresentation, i.e., on the number of observed appearances in target promoters relative to the expectation from the total appearances in all promoters. −log_10_ *P,* a metric of overrepresentation, is plotted against rank (circles). Eight motifs were chosen for experimental characterization (open circles). (B) Best words grouped into eight candidate motifs. The eight most overrepresented motifs from Fig. 1A, aligned to illustrate common core sequences. The example of each motif chosen for experimental characterization is underlined.

Because words with similar sequences are likely to possess similar biological activity, we considered groups of related words as units in our subsequent analysis. We grouped the dictionary into motifs by performing every possible pairwise alignment between all words, and then clustering words with high mutual alignment scores. A motif may contain two or more words, or just a single word. For a multiword motif, the words defining the motif are similar to one another and share common core sequences (Figure 1B; Table S2). The clustering procedure yielded about 100 motifs, about half of which contain multiple words.

We reasoned that motifs that are likeliest to represent bona fide regulatory elements will be nonrandomly distributed in the genome and appear more often in the UPR target gene promoters than expected by chance. Therefore, we counted the number of times each motif (i.e., a sequence match to any of the words the motif comprises) appeared in the approximately 6,000 promoters in the genome, and computed from this figure the frequency with which each motif would be expected to appear in a promoter if it were distributed randomly throughout the genome. We then counted the number of times the motif was actually found in the 381 target promoters and calculated the probability *P* of this many or more appearances occurring by chance. A small *P* value (high −log_10_ *P*) indicates that the motif is overrepresented relative to the expectation. Figure 1A shows the motifs ranked in order of decreasing overrepresentation, with −log_10_ *P* for each motif plotted against this rank. We chose the eight highest-ranking motifs (open circles) as candidates for experimental testing (Figure 1B), analyzing a single example of each (underlined sequences).

### Experimental Verification of Novel UPREs

To determine whether any of the eight candidate motifs would function as bona fide UPREs, we introduced three tandem repeats of a single representative sequence of each motif into a *lacZ* reporter construct that contains a crippled version of the *CYC1* promoter that is transcriptionally silent in the absence of a UAS (Guarente and Mason 1983). Analogous constructs containing the “classical,” *KAR2*-derived UPRE inserted upstream of the core promoter have been shown to drive transcription of this reporter gene under ER stress (Mori et al. 1992; Cox et al. 1993). As a positive control for UPR-dependent gene expression, we used a construct containing a triple repeat of the *KAR2*-derived UPRE (Cox and Walter 1996). We transformed the resulting plasmids into yeast and assayed for β-galactosidase activity in response to ER stress.

**Figure 2:**
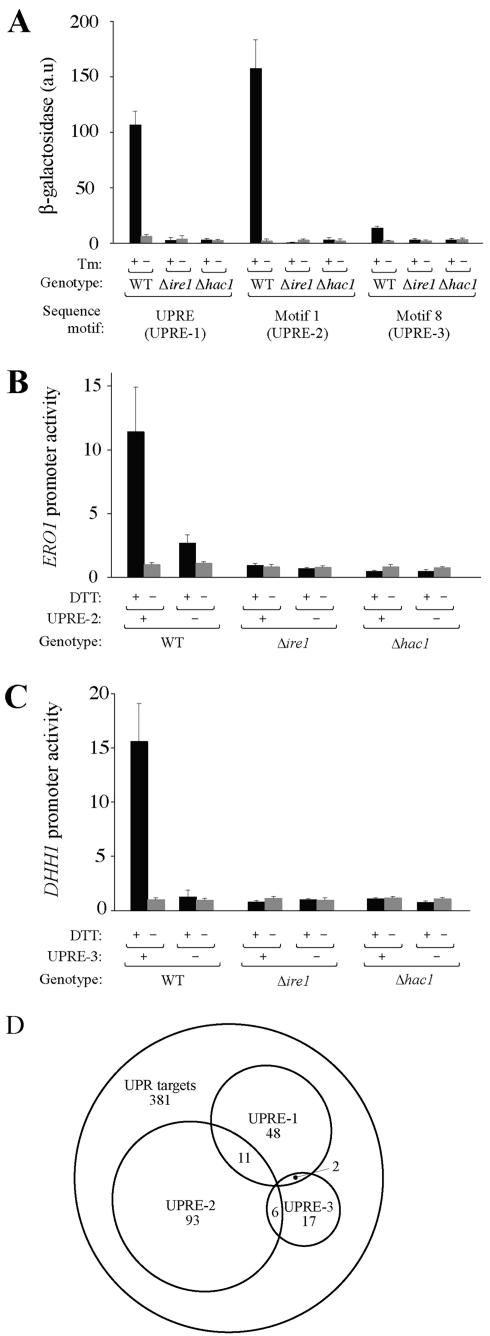
Identification of Two Novel Sequence Motifs Necessary and Sufficient for UPR Activation (A) Motif 1 and Motif 8 are sufficient to confer UPR-responsive transcription on an artificial promoter*.* Single representative sequences of the *KAR2-*derived UPRE and candidate regulatory motifs Motif 1 and Motif 8 were cloned into a crippled promoter driving *lacZ,* transformed into yeast (WT, Δ*ire1,* and Δ*hac1*), and β-galactosidase activity monitored in response to Tm treatment. (B) UPRE-2 (Motif 1) is necessary for UPR-dependent activation of the *ERO1* promoter. lacZ was placed under the control of the WT *ERO1* promoter (+ UPRE-2) or a mutant (− UPRE-2), and β-galactosidase activity monitored in response to DTT treatment. (C) UPRE-3 (Motif 8) is necessary for UPR-dependent activation of the *DHH1* promoter. As in (B), except using the *DHH1* promoter, in which UPRE-3 appears once. (D) Novel motifs explain a greater fraction of UPR target gene activation. Sets of genes whose promoters contain UPR-responsive UASs UPRE-1, UPRE-2, UPRE-3, or a combination, are here depicted in Venn diagram format as subsets of the 381-gene UPR target set.

Of the eight reporter constructs, the two containing Motif 1 and Motif 8 were transcriptionally activated when cells were treated with tunicamycin (Tm) (Figure 2A), or dithiothreitol (DTT) (unpublished data), both inducers of the UPR. The other six motifs showed no activity above baseline (unpublished data). Neither Motif 1 nor Motif 8 showed any activity in the absence of ER stress, and no activation was observed upon UPR induction in either Δ*ire1* or Δ*hac1* strains. Hence, as with the “classical” UPRE, these two motifs are sufficient to confer transcriptional activation upon a promoter in an *IRE1*-*, HAC1*-*,* and ER stress-dependent manner. We therefore conclude that the bioinformatics analysis has identified two novel UPREs present in target gene promoters; hereafter, we refer to Motif 1 and Motif 8 as UPRE-2 and UPRE-3, respectively. Correspondingly, we shall refer to the classical, *KAR2*-derived UPRE as UPRE-1.

To test whether these motifs are also necessary for transcriptional activation, we designed *lacZ* reporter constructs derived from two native promoters in which the motifs appear. We chose for UPRE-2 the promoter of *ERO1,* encoding an ER resident redox protein, and for UPRE-3 the promoter of *DHH1,* encoding an RNA helicase. Both genes are robust targets of the UPR (Travers et al. 2000) and lack a recognizable UPRE-1. First, we verified that the reporters responded to ER stress in a UPR-dependent manner. WT but not Δ*ire1* or Δ*hac1* cells bearing the UPRE-2-containing *ERO1*-promoter-driven reporter expressed higher levels of β-galactosidase after treatment with DTT (Figure 2B, “+ UPRE-2” columns). In a mutant version of this reporter construct, in which the UPRE-2 was ablated and replaced by an unrelated sequence of identical length, inducibility of the *ERO1* promoter was decreased by approximately 4-fold (“− UPRE-2” columns). Similarly, WT but not Δ*ire1* or Δ*hac1* cells bearing the UPRE-3-containing *DHH1*-promoter-driven reporter expressed higher levels of β-galactosidase after treatment with DTT (Figure 2C, “+ UPRE-3” columns); ablation of UPRE-3 from the *DHH1* promoter entirely eliminated induction by ER stress ( “− UPRE-3” columns).

Taken together, the data presented so far indicate that, as with the classical UPRE-1, UPRE-2 and UPRE-3 are both sufficient (Figure 2A) and necessary (Figure 2B and 2C) to confer UPR inducibility on a target promoter. The addition of UPRE-2 and UPRE-3 to the repertoire of UPREs triples the number of genes in the UPR target set whose induction we can explain by invoking the presence of a well-defined UAS (Figure 2D).

### Identification of High-Copy Activators of UPRE-2

The existence of functional *cis*-regulatory elements that differ in sequence from the canonical UPRE-1 suggests that *trans*-activating factors other than Hac1p may bind these elements. Alternatively, Hac1p, alone or accompanied by another factor or factors, may be able to recognize multiple sequences. To distinguish between these possibilities and potentially reveal novel regulatory factors, we attempted to identify genes which, when overexpressed, activate transcription of the UPRE-2 reporter plasmid in the absence of an ER stress signal. The design of this screen recapitulates the approach which identified *HAC1* as a high-copy activator of the UPRE-1 (Cox and Walter 1996).

We transformed a strain bearing the UPRE-2-*lacZ* reporter with a 2-μm-plasmid-derived ( high-copy) genomic DNA library (Miller et al. 1984). A Δ*ire1* strain was used in order to focus the screen on genes acting downstream of *IRE1.* Use of the Δ*ire1* strain also avoided a background of false positives resulting from library plasmids encoding secretory proteins whose overexpression might activate Ire1p. Transformants were plated on synthetic defined media and, after appearance of colonies, overlaid with soft agar containing the β-galactosidase substrate X-gal. Colonies that turned significantly more blue than control (untransformed) colonies were recovered and rescreened by the same assay. Plasmids from positively rescreened clones were retransformed into the Δ*ire1* UPRE-2-*lacZ* strain to verify plasmid linkage of the activator phenotype.

We screened a total of 112,000 transformants, representing a predicted genomic coverage of approximately 50x. Thirty-eight positive transformants passed through repetition and plasmid linkage tests, and 18 of these stably maintained the activator phenotype over many generations. Positive plasmids fell into two classes, as defined by the minimal region of overlap of their insert sequences. One class of inserts (ten plasmids) shared the *IRE1* locus and surrounding sequences; *IRE1* has been previously shown to be activated by overexpression and is a high-copy activator of UPRE-1 (Cox et al. 1993). Recovery of this locus demonstrates that the screen was able to capture genes of physiological relevance to the pathway.

**Figure 3:**
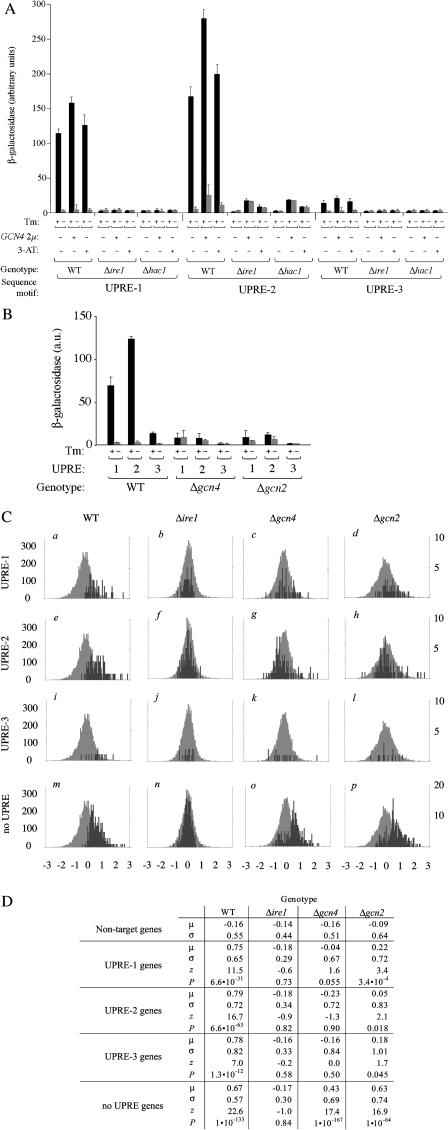
*GCN4* Encodes a Novel Transcription Factor in the UPR (A) Overexpression of *GCN4* is sufficient for activation of UPRE-2, but not UPRE-1 or UPRE-3. UPRE-driven transcriptional activity as a function of Gcn4p levels, elevated either as a result of overexpression (+ *GCN4–2μ*) or amino acid starvation (+ 3-AT), in the presence or absence of ER stress (Tm). (B) *GCN4* and *GCN2* are necessary for ER stress-dependent activation of UPRE-1 and UPRE-2. UPRE-driven transcriptional activity as a function of *GCN4* pathway genes (WT, Δ*gcn4,* and Δ*gcn2*) in the presence or absence of ER stress (Tm). (C) *GCN4* and *GCN2* are required for UPR-dependent transcriptional activation of a subset of target genes. Fold changes in mRNA levels were determined by microarray for DTT-treated vs. -untreated WT, Δ*ire1,* Δ*gcn4,* and Δ*gcn2* strains (columns). Histograms show distribution of log_2_-fold changes for non-UPR target genes (light bars) and for UPR target genes (dark bars), which contain UPRE-1, UPRE-2, UPRE-3, or still unidentified UPREs (rows) in their promoters. (D) Target gene regulation differs significantly in WT and *Δgcn4/Δgcn2* mutants. Means *(μ)* and standard deviations *(σ)* for log_2_-fold change in gene expression for non-UPR target genes, and for genes that fall inside the UPR target gene set and contain UPRE-1, UPRE-2, or UPRE-3 in their promoters. Z statistic *(z)* and *P* value *(P)*: higher *z* reflects a greater difference between the distribution for UPRE-containing target genes and nontarget genes; lower *P* indicates a more highly significant difference. For detailed calculations, see Materials and Methods.

The second class of positive inserts (eight plasmids) shared the *GCN4* locus. *GCN4* encodes a bZIP transcription factor, which has been well-characterized as a component of the cellular response to amino acid starvation and other stresses (Natarajan et al. 2001; reviewed in Hinnebusch 1997) but has not been previously demonstrated to play a role in the UPR. We constructed a 2-μm plasmid bearing only *GCN4,* transformed it into WT, Δ*ire1,* and Δ*hac1* strains carrying UPRE-1-*lacZ,* UPRE-2-*lacZ,* and UPRE-3-*lacZ* reporters, and assayed for β-galactosidase activity (Figure 3A). *GCN4* overexpression stimulated UPRE-2-driven reporter activity in all three genotypes ( “+ GCN4 2μ” columns), indicating that overexpression of *GCN4* is sufficient to stimulate transcription from the UPRE-2-driven reporter gene in the absence of ER stress, Ire1p activity, or Hac1p production. We also starved cells for histidine by administering 3-aminotriazole (3-AT), which induces translation of Gcn4p (Albrecht et al. 1998). As when cells expressed high levels of *GCN4,* amino acid starved cells exhibited a significant increase of UPRE-2 transcription in the absence of ER stress ( “+3-AT, −Tm” columns). *GCN4* overexpression alone did not activate transcription from either UPRE-1 or UPRE-3 reporter genes, emphasizing that these motifs are not synonymous with UPRE-2.

### *GCN4* and *GCN2* Are Required for Activation of All Three UPREs

Having demonstrated that *GCN4* overexpression is sufficient to activate transcription from a UPRE-2 reporter, we next asked whether *GCN4* is also necessary to activate transcription in response to ER stress. We deleted *GCN4* from strains bearing UPRE-1, UPRE-2, and UPRE-3 reporter constructs and assayed β-galactosidase activity in response to UPR activation. Upon UPR induction, *HAC1* mRNA was spliced normally, and Hac1p was produced at WT levels in Δ*gcn4* mutants (unpublished data). However, Δ*gcn4* cells failed to induce transcription, not only of the UPRE-2-driven reporter but also of the UPRE-1- and UPRE-3-driven reporters (Figure 3B). Hence we conclude that *GCN4* is required for ER stress responsiveness of all three UPREs.

Consistent with the genetic requirement for *GCN4,* high levels of Gcn4p potentiate transcription from all UPREs when the UPR is activated. *GCN4* overexpression increases the level of reporter activation in WT cells when the UPR is induced (Figure 3A, compare “*GCN4* +Tm” to “*GCN4 −*Tm” data), suggesting that *GCN4* activity is limiting for UPR-dependent transcription from all three UPREs. Similarly, stimulation of Gcn4p production by amino acid starvation also increases the magnitude of the transcriptional response (Figure 3A, “+3-AT, +Tm” data).

In its role in the transcriptional response to amino acid starvation, *GCN4* is activated at the translational level. Uncharged tRNAs are detected by the kinase Gcn2p, which phosphorylates initiation factor 2α (eIF-2α); when eIF-2α is phosphorylated, scanning ribosomes fail to initiate at upstream ORFs encoded by the *GCN4* 5′ UTR and are able to initiate translation at the *GCN4* ORF itself (Hinnebusch 1997). We therefore asked whether *GCN2* is also required for *GCN4* activity in the context of the UPR. As with Δ*gcn4* cells, Δ*gcn2* strains were also unable to mount a transcriptional response from any of the reporter constructs (Figure 3B).

Given that *GCN4* and *GCN2* are necessary for ER stress-dependent transcription in an artificial promoter context, we next asked whether these genes are required for upregulation of the target genes of the UPR. To this end, we measured steady-state mRNA levels by microarray hybridization, comparing WT, Δ*ire1,* Δ*gcn4,* and Δ*gcn2* cells treated with DTT for 30 min (by which time the UPR is qualitatively complete; Travers et al. 2000) to untreated samples of the same genotype. WT cells were taken as a positive control for UPR induction, and Δ*ire1* cells as a negative control. Fold change in expression of a given gene was computed as the ratio of mRNA level in the treated sample to the level in an untreated sample of the same genotype.

In our analysis, we considered five subsets of genes: the sets of UPR target genes containing a UPRE-1, UPRE-2, or UPRE-3 in their promoter, the set of UPR target genes without an identified UPRE in their promoters (“no UPRE”), and the set of genes previously identified as UPR-independent (“nontargets”) (Travers et al. 2000). The distributions of the log_2_-fold changes for each subset of genes in each genotype relative to the set of nontarget genes are illustrated in Figure 3C. For each gene set in each genotype, we determined the difference between the distributions of log_2_-fold changes in UPRE target genes and those in nontarget genes. The statistical significance of these differences is represented by the *z* scores and *P* values enumerated in Figure 3D; higher *z* and lower *P* indicate a greater difference between distributions and higher significance (for details see Materials and Methods).

The majority of the genes in the nontarget set (Figure 3C, all histograms, light bars) are not differentially regulated by ER stress in the WT and mutant strains. As previously shown, however, genes of the UPR target set are significantly more upregulated in the WT than in Δ*ire1* cells (Figure 3C, compare dark bars versus light bars between histograms *a* and *b, e* and *f, i* and *j,* and *m* and *n*). This is the case both for target genes bearing any UPRE in the promoter (Figure 3C, histograms *a–l*) as well as the remainder of the target set for which a UPRE has not been identified (Figure 3C, histograms *m–p*). For those genes with an identified UPRE in their promoters, expression patterns in both Δ*gcn4* (Figure 3C, histograms *c, g,* and *k*) and Δ*gcn2* mutants (Figure 3C, histograms *d, h,* and *l*) show trends similar to those in Δ*ire1*. In both mutants, the sets of genes whose promoters contain a UPRE are significantly less upregulated relative to their induction in the WT. Some UPR target genes exhibit residual upregulation in Δ*gcn4* and Δ*gcn2,* suggesting that these promoters have only a partial requirement for *GCN4*/*GCN2*. This effect is most prominent for genes containing UPRE-1 in the Δ*gcn2* mutant (Figure 3C, histogram *j*), where the residual induction crosses the threshold into marginal statistical significance (Figure 3D, “Δ*gcn2,* UPRE-1”; *p* = 3.4 × 10^−4^); it is possible that the residual levels of Gcn4p present in a Δ*gcn2* mutant are sufficient to allow UPR transcription from these promoters, or alternatively that UPRE-1 promoters are relatively less sensitive to Gcn4p levels (and concomitantly, relatively more reliant on Hac1p) for induction (see Discussion). In contrast, induction of the “no UPRE” genes is quite high in Δ*gcn4* and Δ*gcn2* cells (Figure 3C, histograms *o* and *p* versus *m*). As a population, these genes are not significantly less upregulated in the mutants than in the WT. It would appear that the UPREs identified to date define a special subset of UPR target genes that are responsive not only to *IRE1* and *HAC1* but that are particularly sensitive to the *GCN4*/*GCN2* branch of the pathway.

Overall, in both Δ*gcn4* and Δ*gcn2* mutants, the pattern of gene regulation during the UPR is similar to that in the Δ*ire1* mutant: Mean fold changes of UPRE-containing target genes are lower in these mutants than in the WT. We conclude that *GCN4* and *GCN2* play a broad role in the UPR, contributing significantly to the upregulation of a large subset of UPR target genes.

### Gcn4p Is Upregulated in Response to ER Stress

Given the requirement for *GCN4* in UPR-dependent transcription, and in particular the observation that Gcn4p appears to be limiting for the magnitude of the transcriptional response (Figure 3A), we asked next whether Gcn4p levels would be subject to posttranscriptional regulation under conditions of ER stress. We discounted the possibility that *GCN4* would be regulated at the transcriptional level, as our previous studies showed that *GCN4* mRNA levels are unchanged over the course of the UPR (Travers et al. 2000).

**Figure 4:**
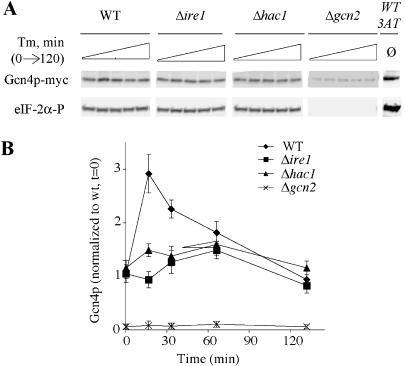
Gcn4p Protein Levels Are Upregulated during the UPR (A) Gcn4p levels, but not eIF-2α phosphorylation, rise under ER stress in a UPR-dependent manner. Cells bearing a C-terminally *myc*-tagged allele of *GCN4* were treated with Tm for 0, 15, 30, 60, or 120 min. Western blots probed with anti-myc recognizing Gcn4p-myc (top panels) or phospho-specific anti-eIF-2α antibody (bottom panel). Gcn4p blot for the Δ*gcn2* mutant is 5x overexposed so that the bands are visible. (B) Quantitation of the Gcn4p-myc protein levels in Figure 4A. Data reflect an average of four experiments, normalized against Gcn4p levels in the WT *t* = 0 samples.

We constructed strains expressing a C-terminally myc-epitope-tagged allele of Gcn4p, which complements the slow growth phenotype of a Δ*gcn4* mutant and is inducible by amino acid starvation resulting from 3-AT treatment (Figure 4A, “Gcn4p” lanes, compare “wt, +3-AT” to “wt, 0 min”). Over a time course of UPR induction, Gcn4p-myc levels exhibited a transient increase of 2.5-fold, peaking after 15 min and gradually decaying to uninduced levels after 60–120 min (Figure 4A, “WT” lanes; quantitated in Figure 4B). This temporary increase in Gcn4p was not observed in UPR-deficient mutants: neither Δ*ire1* nor Δ*hac1* mutants exhibited increased levels of Gcn4p over the time course of UPR induction.

In the context of other stress responses (e.g., amino acid starvation), Gcn4p levels are regulated via phosphorylation of eIF-2α by Gcn2p (Dever et al. 1993; Hinnebusch 1993; Diallinas and Thireos 1994). Because *GCN2* is required for induction of UPR-dependent transcription, we asked whether *GCN2* was required for the rise in Gcn4p levels we observed during Tm treatment. Basal levels of Gcn4p are low in a Δ*gcn2* strain (less than 10% of WT), as previously reported (Hinnebusch 1993; Tavernarakis and Thireos 1996). We observed no increase in Gcn4p levels during the time course in this mutant (Figure 4B).

These data are consistent with two possibilities: first, that Gcn2p is responsible for both basal levels of Gcn4p and its induction upon ER stress; or second, that Gcn2p is responsible only for maintaining basal levels of Gcn4p, while another pathway mediated by Ire1p/Hac1p further elevates Gcn4p levels during the UPR. If Gcn2p is responsible for upregulation of Gcn4p during the UPR, we should observe a concomitant increase in the level of eIF-2α phosphorylation. We did not observe such an increase (Figure 4A, “eIF-2α-P” lanes), which is consistent with the idea that Gcn2p's role in the UPR is primarily to maintain basal levels of Gcn4p, not to upregulate Gcn4p via increased eIF-2α phosphorylation. Other workers have observed a transient increase in phospho-eIF-2α under Tm treatment (Cherkasova and Hinnebusch 2003). It is possible that strain differences or the significantly greater doses of Tm used in the previous study (4 and 20 μg/ml versus our 1 μg/ml) explain this disparity. Consistent with our findings, Cherkasova and Hinnebusch (2003) predict derepression of *GCN4* by ER stress mediated by increased phospho-eIF-2α. Here, we observe increased Gcn4p levels under ER stress conditions even when phospho-eIF-2α levels are not detectably altered.

### Epistasis of *HAC1* and *GCN4*

*GCN4* plays an essential role in the UPR, with a knockout phenotype closely resembling that of Δ*ire1* and Δ*hac1:* the absence of any of these genes prevents transcriptional activation by ER stress. This observation could be a consequence of one of several different mechanisms: Gcn4p might act upstream or downstream of Hac1p in the same linear pathway, or act in a parallel pathway that converges at target promoters. Two lines of evidence from data already introduced argue that Gcn4p does not act upstream of Hac1p. First, *GCN4* overexpression is sufficient to activate transcription from UPRE-2 in a Δ*hac1* mutant (see Figure 3A), indicating that Gcn4p's influence on target promoters can occur by a Hac1p-independent mechanism. Second, the transient upregulation of Gcn4p levels observed under ER stress is absent in the Δ*hac1* mutant (see Figure 4A), indicating that Hac1p levels determine Gcn4p levels.

**Figure 5:**
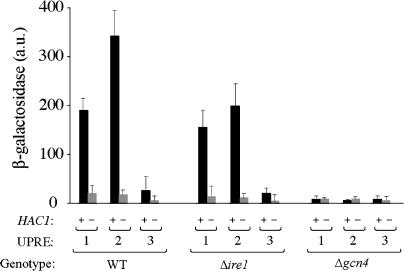
*GCN4* Acts with or Downstream of *HAC1* UPRE reporter activity as a function of Hac1p expression and UPR pathway genes. To express Hac1p in the absence of ER stress, we used an intron-less allele of *HAC1,* which is constitutively translated.

Further evidence that Gcn4p does not act upstream of Hac1p is provided by the observation that expression of Hac1p cannot activate transcription in a Δ*gcn4* mutant (Figure 5). In a WT cell, expression of Hac1p produced from a *HAC1* gene lacking the intron is sufficient to activate transcription from the UPRE-1 (Cox and Walter 1996; Figure 5, “UPRE-1” columns). Constitutive expression of Hac1p is also sufficient to activate UPRE-2, and to a lesser extent UPRE-3, in the absence of ER stress (Figure 5, “WT, +Hac1p” columns) and in the absence of Ire1p (Figure 5, “Δ*ire1*, +Hac1p” columns). In the absence of *GCN4,* however, the constitutive expression of Hac1p does not activate transcription from any of the three reporter constructs (Figure 5, “Δ*gcn4,* +Hac1p” columns), suggesting that Hac1p's function at promoters containing any one of the three UPREs requires the presence of Gcn4p. Thus, Gcn4p must act at the same point as or downstream of Hac1p. Following the same line of reasoning, for UPRE-1 and UPRE-3, *GCN4* overexpression alone is insufficient to activate transcription in the absence of *HAC1* (e.g., see Figure 3A, Δ*hac1* mutants), indicating that at UPRE-containing promoters Hac1p must act at the same point as or downstream of Gcn4p. Thus, the observations enumerated here are consistent with the interpretation that Gcn4p and Hac1p act together at target gene promoters.

### A Gcn4p/Hac1p Complex Binds Both the UPRE-1 and UPRE-2

**Figure 6:**
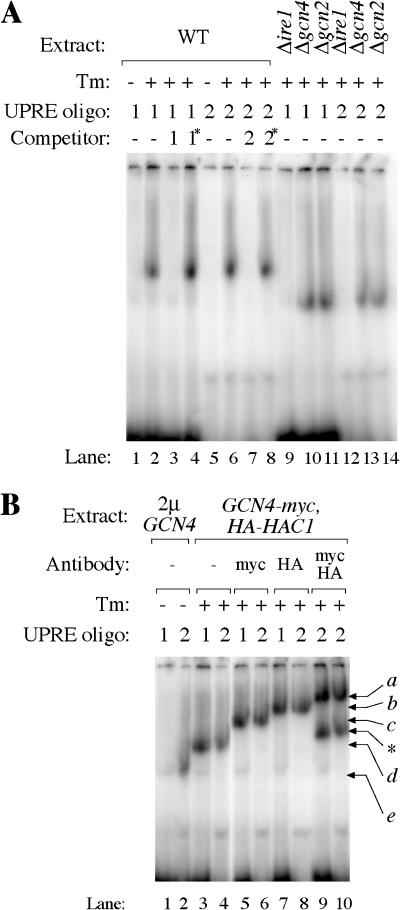
Hac1p and Gcn4p Directly Interact with UPRE-1 and UPRE-2 ^32^P-labeled oligos bearing either UPRE-1 or UPRE-2 promoter were incubated with crude cell extracts, and subjected to nondenaturing polyacrylamide gel electrophoresis. (A) Extract: Samples were of the WT, or bore deletions in *IRE1*Δ*ire1), GCN4 (*Δ*gcn4),* or *GCN2 (*Δ*gcn2),* and were treated with Tm (+) or mock treated (−). Labeled oligos contained either UPRE-1 (1) or UPRE-2 (2). Binding reactions were incubated with no unlabeled competitor (−) or with 100x excess of unlabeled WT UPRE-1 (1), an inactive mutant version of UPRE-1 (1*), UPRE-2 (2), or an inactive mutant version of UPRE-2 (2*). (B) Extract: Samples from a strain overexpressing *GCN4* (2μ-*GCN4;* lanes 1 and 2) or from a strain expressing myc-tagged Gcn4p and HA-tagged Hac1p *(GCN4-myc* and *HA-HAC1).* Binding reactions were incubated with no antibody (−), anti-myc recognizing Gcn4p-myc (myc), anti-HA recognizing HA-Hac1p (HA), or both antibodies simultaneously (myc/HA). Bands represent the following: *a,* Gcn4p + Hac1p + anti-myc + anti-HA; *b,* Gcn4p + Hac1p + anti-HA; *c,* Gcn4p + Hac1p + anti-myc; *d,* Gcn4p + Hac1p; *e,* Gcn4p. *, an unidentified band that appears only when extracts include both Gcn4-myc and HA-Hac1p and when both antibodies are included in the binding reaction.

To explore this possibility directly, we performed gel-retardation assays with the UPRE-1-containing segment of the *KAR2* promoter (oligo 1), used in previous experiments demonstrating direct binding of Hac1p to UPRE-1 (Cox and Walter 1996), and the UPRE-2-containing segment of the *ERO1* promoter (oligo 2). ^32^P-labeled oligonucleotides were incubated with cell extracts and subjected to native (nondenaturing) polyacrylamide gel electrophoresis, and visualized by autoradiography (Figure 6).

As previously observed, oligo 1's mobility was retarded when incubated with crude extracts from UPR-induced cells, but not extracts from untreated cells (Figure 6A; compare lane 2 to lane 1). Likewise, oligo 2 was specifically shifted by extracts from UPR-induced cells (compare lane 6 to lane 5). The binding activity is specific: for both oligos, the mobility shift was competed out by 100-fold excess of an unlabeled identical sequence (lanes 3 and 7) but not by a transcriptionally inactive point mutant of the same sequence (lanes 4 and 8). The binding activity is dependent on an intact UPR. No gel retardation was observed for either sequence in an Δ*ire1* mutant (lanes 9 and 12), in which Hac1p cannot be synthesized. Likewise, in both Δ*gcn4* and Δ*gcn2* mutants, the binding activity observed in WT cells was absent. In both Δ*gcn4* and Δ*gcn2* mutants, however, a faster migrating complex appeared, which likely represents Hac1p alone binding the oligos (lanes 10, 11, 13, and 14).

To demonstrate Gcn4p and Hac1p binding conclusively, we performed supershift analyses of the WT complex by addition of antibodies to either protein. We constructed a strain expressing both HA-epitope-tagged Hac1p and myc-tagged Gcn4p. Extracts from Tm-treated cells were incubated with antibodies against either or both tagged proteins. Antibodies recognizing either the tagged Gcn4p-myc (Figure 6B, lanes 5 and 6) or HA-Hac1p (lanes 7 and 8) supershifted the bound complex to different extents (compare lanes 7 and 8 to lanes 3 and 4). Hence, both Gcn4p and Hac1p can bind to sequences containing UPRE-1 and UPRE-2. Addition of both antibodies to the same binding reaction resulted in an ultrashifted band, migrating more slowly than the bands in either of the single antibody reactions (lanes 9 and 10). If Hac1p and Gcn4p bound DNA in distinct, separate complexes, we would expect to see two bands of identical mobility to those seen in lanes 5–8. We conclude that the mobility-shifted complex observed in UPR-induced WT cells therefore must contain both transcription factors, since no ultrashift would occur if the proteins were bound to separate complexes, and that Hac1p and Gcn4p act together at the same location to activate transcription upon UPR induction. (Similar gel-shift experiments performed with an oligonucleotide representative of UPRE-3 failed, indicating that transcription factor binding may be of reduced affinity at this sequence. This interpretation is consistent with the overall lower activity of the UPRE-3 reporter constructs (see Figure 2A).

Further evidence that Gcn4p can bind UPRE-2 is provided by the observation that overexpression of *GCN4* alone in an otherwise WT cell, in the absence of ER stress, resulted in a mobility shift for oligo 2 (Figure 6B, lane 2). This complex migrated faster than the WT complex (e.g., Figure 6B, lane 4). Because the extract was made from untreated cells, no Hac1p was present, indicating that the complex contains Gcn4p alone. The *GCN4*-dependent shift is not observed for oligo 1, consistent with observations above that Gcn4p overproduction is sufficient to activate transcription of a UPRE-2 reporter but not a UPRE-1 reporter (see Figure 3A). Reciprocally, Hac1p is present in the Δ*gcn4* and Δ*gcn2* mutants, but Gcn4p is absent; it therefore seems likely that the faster migrating bands in Δ*gcn4*/Δ*gcn2* mutants (Figure 6A, lanes 10, 11, 13, and 14) represent oligonucleotides bound to Hac1p alone.

## Discussion

### Identification of Novel UASs

Beginning only with the set of genes induced by the UPR and the promoter sequences of all genes in the genome, we computationally identified candidate motifs that obeyed the statistical properties we would expect of regulatory sequences, i.e., high frequency in UPR target promoters, and enrichment in the target promoters relative to the rest of the promoters in the genome. Two of these motifs, UPRE-2 and UPRE-3, are both necessary and sufficient to confer ER stress responsiveness in an *IRE1*- and *HAC1*-dependent manner on promoters which contain them. These novel sequences are activated under the same conditions as UPRE-1. Functional non-synonymy of these sequences, however, is illustrated by the activation of UPRE-2 by *GCN4* overexpression alone, a condition under which UPRE-1 and UPRE-3 are silent, and by the quantitative difference with which the motifs respond to UPR activation (UPRE-2 > UPRE-1 > UPRE-3). Although the two new UPRE sequences look at first glance entirely different from the well-characterized UPRE-1, one of them may share “half-site” similarity: UPRE-2 has a three base identity with UPRE-1 at the 3′ end (TACGTG versus CAGNGTG); whether these bases make equivalent contacts with the bound transcription factors remains to be determined. Taken together, the sequence diversity of the motifs conferring similar transcriptional control upon binding of the same transcriptional activators illustrates the difficulty of predicting biological regulation from promoter sequences alone, even if binding sites in one context are well defined experimentally.

The identification of these novel sequences allows a greater proportion of UPR target gene regulation to be explained within the paradigm of modular transcriptional control, i.e., in which a “portable” sequence module (a UAS) located within a promoter confers pathway responsiveness on the gene in question. The two novel motifs described triple the number of target genes whose regulation can be described in terms of a modular control mechanism, thus adding significantly to the repertoire of *cis*-acting elements known to act in the UPR.

And yet, the resulting description of UPR transcription remains incomplete, as approximately 50% of the target genes still lack a recognizable UPREs. It may be that more biologically active motifs exist among the 109 motifs that emerged from the overrepresentation analysis, as many of the untested motifs are overrepresented relative to chance in the UPR target set by many orders of magnitude. For the eight motifs tested, we tested whether a motif was necessary for promoter induction only if it had already been shown to be sufficient in the artificial promoter system. Because of this experimental approach, it remains possible that some motifs not found to be sufficient are dependent for their activity on some contextual parameter (e.g., particular nearby flanking sequences). Thus it may be that some UPREs are not generally portable to other contexts, but are nonetheless necessary for UPR responsiveness of the native promoters in which they reside. Also, particularly rare motifs would have been omitted from the dictionary; thus, it is possible that complementary computational approaches might allow detection of uncommon motifs that this analysis missed. Finally, some UASs may remain ultimately undiscoverable within the paradigm of modular regulation. Motifs that are particularly sensitive to chromatin structure or position relative to the transcription initiation site would not be detected by an approach that neglected these parameters.

It might be argued that the approach here enjoys no relative advantage over testing random oligonucleotides from UPR promoters. If every sequence from each target promoter were to be tested for activity, it is possible that additional elements not revealed by the bioinformatic approach would be discovered. For example, the residual upregulation of *ERO1* after removal of UPRE-2 (see Figure 2B) suggests that at least one cryptic element exists in that promoter. On the other hand, the *DHH1* promoter shows no residual upregulation after removal of UPRE-3 (see Figure 2C). If the average number of sites (candidate plus cryptic) per promoter is similar (1–2) throughout the target gene set, our computational approach represents a highly efficient means of identifying a subset of regulatory motifs. On the other hand, if the average is significantly higher, it is possible that testing random subsequences of target promoters would also be efficient. From the small number of promoters we studied in depth, it is not possible to calculate a meaningful upper bound for the average number of undiscovered regulatory sites per promoter. Nonetheless, within the sample size of our study, the yield of active regulatory sites per candidate tested (two of eight) is much higher than any reasonable a priori estimate of the density of regulatory elements in the UPR target promoters.

One indication of a possible shortcoming of our computational approach is the finding that the probabilistic segmentation did not return the classical UPRE-1 as a significant “word,” i.e., the approach failed to generate a comprehensive list of all known active UPREs. The absence of UPRE-1 from the dictionary indicates that no sequence matching the experimentally defined degenerate consensus CAGNGTG is intrinsically overrepresented in the target promoters, i.e., this motif does not occur in the “text” of target gene promoters with a higher frequency than that with which its component subsequences would appear together by chance. Neither is this sequence overrepresented in the target promoter set relative to the promoters of the nontarget genes. The motif CAGNGTG has an overrepresentation score −log_10_ *P* of 0.37, far beneath the enrichment of any of the 109 motifs assembled from dictionary words (see Figure 1A). Hence, among genes that possess a UPRE-1 in their promoters, there are more instances of unresponsiveness to the UPR than instances of regulation, even though UPRE-1 has been experimentally demonstrated to be necessary and sufficient for upregulation in response to ER stress.

**Figure 7:**
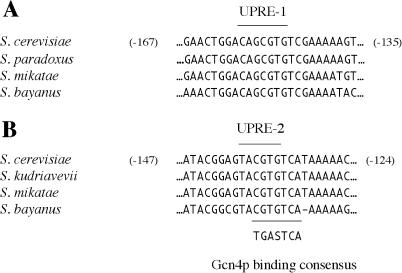
Multiple Alignment of UPRE-1 and UPRE-2 from Three Budding Yeasts Alignment of partial promoter sequences from S. cerevisiae and homologous sequences in related yeasts. Numerical coordinates reflect the distance from the first nucleotide of the initiation codon in the S. cerevisiae promoter. (A) A segment of the *KAR2/YJL034W* promoter and homologs. The core sequence of UPRE-1 is indicated. (B) A segment of the *ERO1*/*YML130C* promoter and homologs. The core sequence of UPRE-2 is indicated (above). The consensus binding site of Gcn4p is aligned (below).

A plausible resolution to this paradox may be that the UPRE-1 is heavily dependent on context. The experiments that defined the key core nucleotides proceeded by single point mutation at each position while holding constant the identity of all other nucleotides from the source 22-bp stretch of the *KAR2* promoter; thus the seven-nucleotide “core sequence” may only specify those bases which are necessary for activity, but not define a module which is generally functional outside its original context of flanking sequence. If this were the case, we would not expect to recover UPRE-1 in a bioinformatic analysis of all target genes. Indeed, alignment of the *KAR2* promoter from S. cerevisiae and three related budding yeasts reveals that UPRE-1 lies in the middle of a highly conserved 21-bp sequence which is 100% identical across three of the species (Figure 7A). This conserved stretch may represent a context that is essential for the transcriptional function of the core sequence. We speculate that recognition of the extended context may be performed by Hac1p without the collaboration of Gcn4p, as suggested by the observation that promoters which contain a UPRE are more dependent on *GCN4*/*GCN2* than are those genes in which a short modular UAS has not been identified (see Figure 3C, histograms *o* and *p*).

Despite these qualifications, the approach has successfully uncovered novel information about how the UPR is regulated. The appealing aspect of the strategy described here is that such studies are not limited to the UPR but can be generally employed in the study of any transcriptional response in any organism for which promoter sequences for all genes are known and in which the comprehensive genomic output of the response can be measured by expression profiling. The sole requirement of the probabilistic segmentation/overrepresentation computations is that a partition of the genome (into “target genes” and “nontarget genes”) be made on the basis of some meaningful difference in expression levels under the conditions of interest; the analysis thereafter proceeds by comparing the distribution of candidate motifs in the target gene set and the remainder of the genome. Further refinement of the mathematical tools therefore promises to be of invaluable help in our quest for a comprehensive understanding of the logic and complex interactions of transcriptional programs in eukaryotic cells.

### *GCN4* Is an Essential Transcription Factor of the UPR

The overexpression screen for activators of UPRE-2 revealed a role for the transcription factor Gcn4p, which we show to be required not only for activity of UPRE-2 but for all three known UPREs. Gcn4p and its upstream activator Gcn2p thus join Ire1p, Hac1p, and Rlg1p in the list of essential players in the yeast UPR.

*GCN4* encodes a well-characterized transcription factor acting in several distinct stress responses including amino acid starvation, glucose limitation, and ultraviolet irradiation (Hinnebusch 1997; Yang et al. 2000; Natarajan et al. 2001; Stitzel et al. 2001), but has not previously been demonstrated to play any role in the UPR. Here, we demonstrate that *GCN4* is required for normal induction of UPR transcription, both in the context of artificial promoters containing any of the known UPREs and in the context of the native promoters of most target genes. *GCN2,* a gene implicated in regulating *GCN4* in other stress responses, is similarly required for a normal UPR, perhaps because *GCN2* function is required to maintain the basal level of Gcn4p in a cell even under normal growth conditions.

Our gel-mobility shift studies demonstrate a direct physical association between Hac1p and Gcn4p and the sequence motifs UPRE-1 and UPRE-2. Gcn4p and Hac1p are bZIP proteins, a family whose members bind DNA as dimers (Ransone et al. 1993; Hsu et al. 1994). It therefore seems likely that Gcn4p and Hac1p stimulate transcription by binding promoter DNA as a heterodimer, although we cannot rule out higher order complexes.

The promoter sequences UPRE-1 and UPRE-2 have identical genetic requirements for activation, but their behavior in response to genetic perturbations is not strictly identical. UPRE-2 can be activated by high levels of *GCN4* alone (see Figure 3A), but UPRE-1 cannot. This can be explained by the binding studies, which demonstrate that UPRE-2 (but not UPRE-1) can bind Gcn4p in the absence of Hac1p (see Figure 6B, lanes 1 and 2); indeed, Gcn4p is known to bind DNA as a monomer as well as a dimer (Cranz et al. 2004) and can bind DNA sequences containing even a consensus half-site (Hollenbeck and Oakley 2000).

The basis for this differential affinity for Gcn4p is strongly suggested by a refined consensus sequence for UPRE-2, and is illustrated by multiple species alignment of the *ERO1* promoter (Figure 7B). We searched for examples of UPRE-2 core sequences that were conserved in UPR target genes across five yeast species, and extracted core and flanking sequences to derive a generalized consensus (see Materials and Methods). The resulting consensus was revealed to be T(C/T)ACGTGT(C/T)(A/C), which differs from the experimentally established UPRE-1 consensus by two nucleotides essential for activity in the *KAR2* promoter context. The conserved extended context of UPRE-2 in this promoter aligns with a consensus binding site for Gcn4p defined by computational analysis of the set of promoters that bind Gcn4p in a genome-wide chromatin immunoprecipitation assay (analysis by W. Wang and H. Li, unpublished data; chromatin immunoprecipitations in Lee et al. 2002b). Comparison of multiple alignments of the extended contexts of UPRE-1 and UPRE-2 in the *KAR2* and *ERO1* promoters (compare Figure 7A and 7B) reveals that the two sequence contexts share a six-nucleotide segment, CGTGTC. The match between UPRE-2 and the Gcn4p consensus is imperfect (five of seven positions), suggesting that the association with Gcn4p and UPR promoters is not identical to the binding of Gcn4p to its “classical” amino acid starvation targets. Rather, these observations suggest that the proposed Gcn4p/Hac1p heterodimeric complex binds to a composite site, of which UPRE-1 and UPRE-2 represent different forms with stronger relative affinities to Hac1p and Gcn4p, respectively. Such a model would explain the residual upregulation of UPRE-1-containing genes in a Δ*gcn2* mutant (see Figure 3C, histogram *j*), which retains some expression of Gcn4p. In the absence of Hac1p but in the presence of high concentrations of Gcn4p (e.g., when *GCN4* is overexpressed), Gcn4p can bind the UPRE-2 on its own, either as a homodimer or a monomer.

### Upregulation of Gcn4p by ER Stress

The transient upregulation of Gcn4p levels, which we observe upon UPR induction, may therefore serve to increase the transcriptional output of the response, especially early in the response. Most UPR target genes are robustly induced after 15 min of ER stress (Travers et al. 2000); hence, the increase in Gcn4p levels occurs at a time suggestive of a role in the initial response.

Gcn4p itself mediates a broad transcriptional program in response to a diverse set of cellular conditions and stresses (Natarajan et al. 2001). The recruitment of Gcn4p therefore provides an opportunity for crosstalk between regulatory pathways and fine-tuning of the magnitude of the UPR. For example, under amino acid starvation, Gcn4p levels are high relative to the baseline of normal growth. In this state, cells with accumulated unfolded ER protein might wish to upregulate ER-associated protein degradation (one output of the UPR; Casagrande et al. 2000; Friedlander et al. 2000; Travers et al. 2000) beyond the level normally provided by the UPR. Such a mechanism might provide for an additional source of amino acids through protein catabolism. Elevated Gcn4p levels and the concomitant increased induction of UPR target genes would serve this need. This view raises the possibility that those genes that most stringently require *GCN4* for normal UPR induction are those that are most urgently required by the cell under specific conditions, under which UPR is induced *and* Gcn4p levels are high for reasons unrelated to ER stress. The relationship between the cellular stress responses that regulate Gcn4p and the potentiation of UPR transcription will therefore be an important subject for future study.

The mechanism by which *IRE1* and *HAC1* mediate the transient increase in Gcn4p remains to be elucidated. Given that Hac1p and Gcn4p are observed in the same complex with DNA, one intriguing possibility is that association with Hac1p serves to stabilize Gcn4p.

### *GCN4* and the Super-UPR: Two Ways to Modulate the UPR

**Figure 8:**
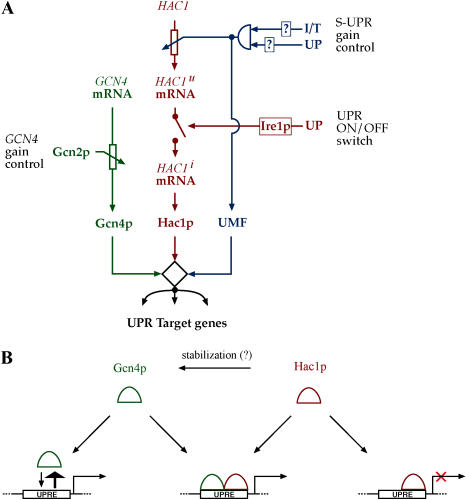
Model of Gcn4p/Hac1p Action in the UPR (A) The expanded circuitry of the UPR*.* The classical UPR (red), the S-UPR (blue), and regulated Gcn4p levels (green) are integrated at target promoters. Transcriptional regulation of *HAC1* mRNA levels, providing one level of gain control, is depicted as a rheostat under supervision of a logical AND gate informed by multiple inputs from the ER. Splicing of *HAC1* mRNA by Ire1p, providing a binary on/off control, is depicted by a switch. Regulation of Gcn4p levels by Gcn2p under changing cellular conditions adds an additional layer of gain control. Together, activity levels of Hac1p, Gcn4p, and the proposed UPR modulatory factor (Leber et al. 2004) collaborate to determine the magnitude of the transcriptional output signal. (B) Mechanism of Gcn4p/Hac1p action at target promoters. In the absence of Hac1p, Gcn4p is present in the cell as a consequence of baseline activity of Gcn2p. At normal concentrations, Gcn4p is unable to bind or activate a target UPRE, but it may bind when Gcn4p levels are elevated. Upon induction of the UPR, Ire1p is activated and Hac1 is synthesized. Hac1p can bind, but not activate, target UPREs. Binding of target DNA by a Gcn4p/Hac1p heterodimer results in a transcriptionally active complex. Gcn4p levels are upregulated under UPR induction, perhaps as a consequence of stabilization by interaction with Hac1p.

We propose a model of UPR transcriptional activation that is illustrated in Figure 8. According to the circuit diagram in Figure 8A, *HAC1* mRNA splicing retains its role as the “switch” that turns the UPR on or off. Gcn4p, whose levels appear to be limiting for the extent of gene regulation, would therefore play a role in setting the “gain” or “volume” of the response, perhaps allowing communication from other stress response pathways in the cell. Such a gain control could serve as an adjunct to the “Super-UPR” (S-UPR) gain control described in the accompanying paper (Leber et al. 2004), whereby an *IRE1-*independent ER surveillance mechanism regulates the transcription of the *HAC1* mRNA in response to compound stresses on the secretory pathway. S-UPR induction proceeds unimpaired in Δ*gcn4* cells, indicating that the S-UPR is mechanistically distinct from the regulation described here (Leber et al. 2004). Whereas the S-UPR monitors conditions of the ER, the *GCN4* branch would integrate information gleaned from the cytosol. Both of these gain controls have the potential to act not only as modulators of the magnitude of the response but also as a tuning dial: UPR targets respond differentially to increased level of *HAC1* during the S-UPR (see the Class 1, 2, and 3 genes in Figure 6 of Leber et al. [2004]). Likewise, different UPR targets exhibit differential dependence on Gcn4p, as is apparent from the variable upregulation of UPR targets in Δ*gcn4* and Δ*gcn2* mutants (see Figure 3C). The observations suggest that increased levels of Gcn4p might serve to differentially upregulate a subset of target genes.

From a mechanistic standpoint, ER stress activates Ire1p, which, through nonconventional splicing, induces Hac1p production (Figure 8B). Hac1p can bind to the known UPREs, but by itself forms a protein–DNA complex that is not competent to upregulate transcription. Gcn4p, which is present at a basal level in cells under normal growth conditions as a result of baseline Gcn2p activity, is unable to bind UPREs in the absence of Hac1p. Gcn4p may bind some UPRE sequences, providing a weak bypass of Hac1p, when it is present at physiologically elevated levels. When Hac1p is produced, Gcn4p is recruited to the UPRE, presumably forming a more stable ternary complex containing promoter DNA, Gcn4p, and Hac1p, and transcription is induced. This ternary complex could be established serially, in which case an inactive Hac1p/UPRE complex would be recognized by Gcn4p, or by recognition of the UPRE by a preformed heterodimer of Gcn4p and Hac1p.

### Conservation between Yeast and Mammalian UPR

Advances in the understanding of the metazoan UPR system has been richly informed by the study of yeast. The elucidation of a role for Gcn4p in the yeast UPR allows us to draw even stronger parallels between the yeast and metazoan systems. In higher eukaryotes, the ER-resident transmembrane kinase PERK is activated by protein unfolding. PERK's cytosolic domain is homologous to Gcn2p and likewise phosphorylates eIF-2α, thereby downregulating general translation but also promoting the selective translation of mRNAs containing upstream ORFs in their 5′ UTR sequences. One of these mRNAs encodes ATF-4, a bZIP transcription factor that represents the metazoan ortholog of Gcn4p. Intriguingly, and in strict analogy to the joint action of Gcn4p and Hac1p proposed here, ATF-4 in metazoan cells collaborates with the Hac1p ortholog XBP-1 to stimulate UPR target gene transcription.

The analogies between the roles of Gcn4p/Hac1p/Gcn2p and ATF-4/XBP-1/PERK suggest that the function of these proteins has been amazingly conserved in the UPR, although the nature of the connections between pathway components may have been adapted over evolutionary time: Yeast does not have an identified PERK ortholog that feeds ER-derived information into the *GCN4* branch of the network. Another parallel concerns S-UPR regulation. In the accompanying paper, Leber et al. (2004) demonstrate that compound secretory stress upregulates *HAC1* mRNA. The mode of modulation of the UPR by the superimposed control of the S-UPR bears a resemblance to the known function of another metazoan transcription factor, ATF-6, which is activated by regulated proteolysis in response to ER stress and in turn upregulates XBP-1 transcription.

In comparison to the metazoan UPR, where multiple ER-resident proteins communicate in a seemingly parallel way with multiple downstream transcription factors, Ire1p and Hac1p remain the central players in the yeast UPR. *GCN4* and the S-UPR provide modulatory functions. Nonetheless, the addition to the repertoire of the yeast UPR effectors of an additional transcription factor (Gcn4p) and of a mechanism for transcriptional regulation of Hac1p (S-UPR; Leber et al. 2004) suggests that the UPR functions as a regulatory network, with its opportunities for crosstalk with other pathways and regulation by cellular state. But most importantly, both the central players and the connectivity of the circuits involved appear to be conserved among eukaryotes and evolutionarily ancient.

## Materials and Methods

### 

#### Computational and quantitative methods

To build the dictionary of putative regulatory elements for UPR target genes, we first extracted the 600-bp upstream regions of all UPR target genes. To get rid of simple repeats unlikely to be regulatory elements (such as AT-rich repeats and transposable elements), we removed exact repeats of lengths 15 bp or longer, and kept the remaining fragments of lengths longer than 50 bp. What remained was the input sequence for the dictionary construction. We used the MobyDick algorithm based on probabilistic segmentation (Bussemaker et al. 2000b) to build a dictionary of putative regulatory elements. MobyDick builds the dictionary by iterating through fitting and testing steps. Starting with the frequencies of single bases, the algorithm finds overrepresented two-nucleotide pairs (testing step), adds them to the dictionary, determines their probabilities by maximizing the likelihood of observing the sequence data (fitting step), and continues to build larger fragments iteratively. Adjustable parameters were as follows: *L,* the maximum word length, was set to 8, and *MaxP,* the probability of seeing at least one false positive at each testing step when all words of length up to *L* are tested, was set to 0.999 (relaxed cutoff). MobyDick generated a dictionary of 328 words. We filtered out words that were too short, appeared in too many copies (such as AT-rich short repeats), or were of low quality (the algorithm calculates a quality factor for each word describing how likely it is that the word can be made by chance from shorter words). With the filters *number_of_copies* < 200, *length* > 4, and *quality_factor* > 0.2, we obtained 201 words.

Using the filtered dictionary, we grouped similar words into motifs using the clustering algorithm CAST (Ben-Dor et al. 1999), as follows: We first computed pairwise alignment scores for all the words in the dictionary, using gapless alignment with a scoring scheme derived from a simple mutation model. The model assumes that a base *x* mutates to any other given base *y* with probability *p*/3, and remains the same base with a probability (1 − *p*). The score for a pair *x–y* is given by the log-odds-ratio of observing the pair under the mutation model versus observing the pair at random. With the choice of *p* = 0.5 (the result is insensitive to the actual *p* value chosen as long as *p* is much smaller than 0.75), a matching pair scores ln(2), and a mismatch scores ln(2/3). We normalized the scores to fall between 0 and 1 by the largest score. We then used the CAST algorithm to group words into clusters, with the threshold parameter set at 0.7 (the lower bound of the normalized score averaged over all pairs in a cluster). This procedure generated 109 motifs.

To test which motifs are significantly overrepresented in the promoters of UPR target genes, we counted for each motif the total number of occurrences in all promoters, and calculated the expected number of occurrences *N_exp_* in the UPR target gene promoters based on the genome-wide frequencies. We then counted the observed number of occurrences *N_obs_* of the motif in the promoters of UPR target genes. We used Poisson statistics to calculate the probability *P* of observing a number of occurrences equal to or greater than *N_obs_* by chance, based on *N_exp_*. The test based on Poisson statistics is a very good approximation of the more rigorous test based on the binomial distribution, where the probability *P* is the probability of seeing a specific instance of the motif in the UPR gene set and the total number of trials *N_t_* is the total number of copies of the motif in the genome. Since *P* is small (0.059) and *N_t_* is large (ranging from approximately ten to approximately 1000) but the product *N_exp_* is finite, the resulting distribution is well approximated by a Poisson distribution with mean = *N_exp_*.

To derive a general consensus for UPRE-2 that includes context information beyond the core motif, we took the five-nucleotide core ACGTG from the Motif 1 alignment (see Figure 1B) and searched the promoters of UPR target genes for the occurrences of this core motif that are conserved across five yeast species. We first took the sequence data for *S. cerevisiae, S. bayanus, S. mikatae, S. paradoxus,* and S. kudriavevii (Cliften et al. 2003; Kellis et al. 2003) and performed multiple sequence alignment on all the orthologous promoters. We then searched for conserved blocks on both strands where ACGTG occurs in all species and is correctly aligned. We found 60 instances of conserved blocks in UPR target gene promoters for which multiple sequence alignment data were available. We then extracted ACGTG plus 10-bp flanking sequences on both side in S. cerevisiae and performed a multiple local sequence alignment of the S. cerevisiae sequences from each of the 60 conserved blocks using the Consensus algorithm (Hertz and Stormo 1999), setting *matrix_width* to 15. The result of the alignment was a position-specific frequency matrix. We derived a consensus sequence from the matrix using the convention by Cavener (1987). The alignment matrix and raw sequence data are available in Table S3.

#### Plasmids and recombinant DNA

DNA manipulations, cloning, and yeast culture were performed as previously described (Sherman et al. 1986; Ausubel 1988; Guthrie and Fink 2002) unless otherwise noted.

UPRE reporter constructs (used in Figures 2A, 3A, 3B, and 5) were based on the plasmid pPW344/pJC104 (Cox et al. 1993), which contains a triple repeat of the *KAR2*-derived UPRE; this plasmid was used as the UPRE-1 reporter in all experiments. To construct UPRE reporters used to test Motifs 1–8, we removed the UPRE-1 repeat from pPW344 by digestion with BglII and XhoI, and replaced it with a triple repeat of a 15-nucleotide sequence encompassing the motif in question and the flanking sequence context. Source sequences were chosen from promoters that exhibited robust induction by the UPR (Travers et al. 2000) and, if possible, did not contain a match to the canonical (*KAR2*-derived) UPRE. Intact promoter reporter constructs (pPW668–pPW671) used in Figure 2B and 2C were also based on plasmid pPW344. Here the promoter of pPW344 (BamHI/BglII fragment) was replaced by a single PCR fragment spanning the approximately 600 nucleotides immediately upstream of either the *ERO1* or *DHH1* initiation codon, or by two fragments spanning the same sequence but with the UPRE motif replaced by a restriction site. The high-copy *GCN4* plasmid (pPW672) used in Figure 3A consists of the region plus1000 nucleotides one either side of the *GCN4* ORF. Source sequence contexts, olignucleotide sequences, and select PCR primers are compiled in Table S4. The plasmids expressing the activated allele of *HAC1* used in Figure 5 (pPW322/pRC43) and the N-terminally HA-tagged allele of *HAC1* (pPW353/pJC316) used in Figure 6B were as previously described (Cox and Walter 1996).

Knockouts of *GCN4* and *GCN2* and the integrated *GCN4-myc* were constructed by PCR cassette/generic primer mutagenesis (Longtine et al. 1998).

#### Yeast strains

All base strains used in this study are enumerated in Table 1. As appropriate, these strains were transformed with plasmids from Table 2 for use in experiments.

**Table 1:**
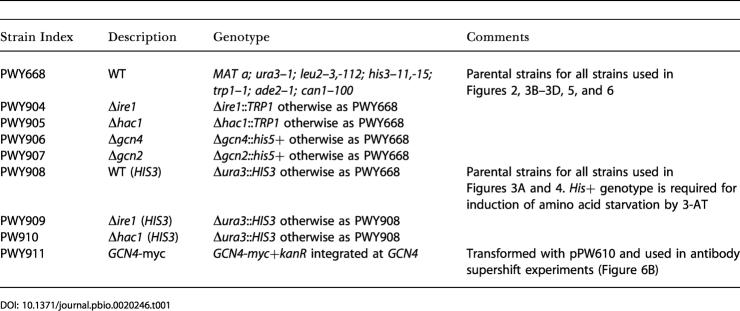
Yeast Strains

**Table 2:**
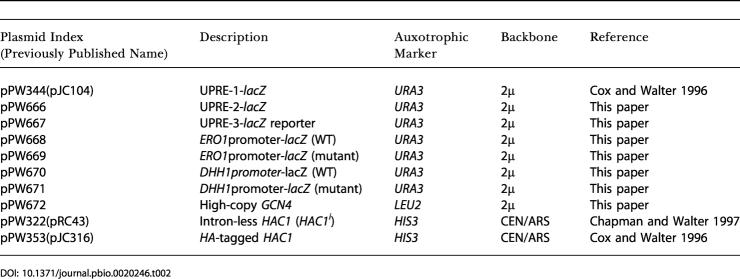
Yeast Plasmids

#### Cell culture and growth conditions

For all experiments, samples were diluted from saturated overnight cultures and regrown to midlog phase (OD600 = 0.5) prior to addition of drug.

DTT (Sigma, St. Louis, Missouri, United States) was added to cultures to a final concentration of 2 mM. Tm (Boehringer Mannheim, Indianapolis, Indiana, United States) was added to cultures to a final concentration of 1 μg/ml. 3-AT (Sigma) was added to cultures to a final concentration of 10 mM. All 3-AT treatments were performed on strains WT for the *HIS3* gene; for histidine-deprived cultures, overnight cultures were washed three times in SD-histidine, then diluted to low density in SD-histidine and grown to midlog phase before the addition of the drug.

To assay β-galactosidase activity on solid growth media, we overlaid plates with buffered soft agar containing X-gal (Sigma) as described previously (Cox and Walter 1996). For liquid cultures, we used a colorimetric ONPG assay (Holley and Yamamoto 1995).

#### Gene expression profiling.

Strains were grown in YPD (pH 5.4) as in Travers et al. (2000) to midlog phase (OD = 0.5) and then either treated with 2 mM DTT or left untreated. RNA was extracted as described by Ruegsegger et al. (2001), and mRNA was purified with a PolyATtract kit (Promega, Madison, Wisconsin, United States).

Microarray analysis used yeast spotted-cDNA ORF arrays printed at the University of California, San Francisco, Core Center for Genomics and Proteomics (http://derisilab.ucsf.edu/more) and was performed as described previously (Carroll et al. 2001). Measurements reported are the average of three independent experiments.

We tested the statistical significance of the induction for the three gene sets (UPRE-1, UPRE-2, and UPRE-3 genes) in four different strains (WT, Δ*ire1,*Δ*gcn4,* and Δ*gcn2*) using a *z*-score scheme. For a given gene set and a given strain, we calculated the average fold induction for genes in the set and compared it to the value for the genome overall. The null hypothesis was that the selected gene set was no different from a randomly selected set (same total number) from the genome overall. Under this hypothesis, the average *μ* has a distribution well approximated by a normal distribution (due to the central limit theorem) with mean *μ_genome_* and standard deviation σ/√(*Nset*), where *N_set_* is the total number of genes in the test set. We computed a *z*-score, *z*=√(*Nset*)(μ−μ*genome*)/σ, which should have a standard normal distribution (zero mean and unit variance) under the null hypothesis. The *P* value was calculated by integrating the standard normal curve from *z* to infinity.

#### Isolation and detection of protein.

Protein preparation, electrophoresis, and Western blotting proceeded as described in the accompanying paper (Leber et al. 2004). Gcn4p-myc (see Figure 5A) was detected using a mouse anti-myc monoclonal antibody (Molecular Probes, Eugene, Oregon, United States); eIF-2α-phosphate was detected by a commercial phospho-specific mouse polyclonal (Upstate Biotechnology, Lake Placid, New York, United States).

#### Gel retardation analysis.

Gel shifts were performed as previously described (Cox and Walter 1996) except that we found it important to elevate the acrylamide concentration to 5% and lower the in-gel glycerol concentration to 4%. UPRE-1 oligo and UPRE-1 mutant are based on sequences previously described (Cox and Walter 1996). UPRE-2 oligo is a fragment of the *ERO1* promoter centered around the UAS. UPRE-2 mutant is a point mutation that does not support transcription in an artificial promoter context (unpublished data). For sequences, see Table S4. Competition experiments used a 100-fold excess of unlabeled oligonucleotide.

## Supporting Information

Table S1 Dictionary of “Words” Compiled by MobyDick This table contains an alphabetical list of the dictionary “words” compiled by the MobyDick algorithm from the “text” comprising the promoters of UPRE target genes. Associated statistics for each word are as follows: *N,* the average number of times the string is delimited as a word among all segmentations of the data; *Xi,* the number of matches of the word anywhere in the text; *p,* the frequency of drawing the word from the dictionary, optimized over all words to give the maximum likelihood of observing the text; *Z* = *p* + *p_s_*, where *p_s_* is the probability with which the word can be made by combining shorter words from the dictionary; *sig* = significance = *Np*/sqrt(*N*[Z − *p*]).
(10 KB TXT).

Table S2 Ranked Listing of the Motifs Assembled by Clustering from the Dictionary Words N_tot_ is the number of times a given motif appeared in the promoters of the genome overall; N_exp_ is the number of times one would expect a given motif to appear in the 381 promoters of UPR target genes if the motif were distributed randomly throughout all promoters; N_obs_ is the number of times a given motif actually appears in the target gene promoters; and −log_10_ *P* is a measure of overrepresentation based on Poisson statistics (*P* is the likelihood that a given observed distribution would occur by chance).
(3 KB TXT).

Table S3 UPRE-Containing Promoter Alignments This table contains CLUSTALW alignments for the *KAR2* and *ERO1* promoters, derived from S. cerevisiae and related budding yeasts. Asterisk indicates 100% conserved residues. Scer, *S. cerevisiae;* Skud, *S. kudriavevii;* Spar, *S. paradoxus;* Smik, *S. mikatae;* Sbay, S. bayanus.
(8 KB TXT).

Table S4 Oligonucleotide Sequences and Cloning Schemes This table contains the sequences of primers and olignonucleotide sequences used in construction of plasmids for this study, as well as oligonucleotide sequences used for probes in the gel-shift analysis.
(38 KB DOC).

### Accession Numbers

The GenBank accession numbers of the gene products discussed in this paper are Dhh1p (NP_010121), Ero1p (NP_013576), Gcn4p (NP_010907), Gcn2p (NP_010569), Hac1p (NP_011946), and Ire1p (NP_116622).

Microarray data can be accessed at the Gene Expression Omnibus (GEO) at the National Center for Biotechnology Information (NCBI) database as platform number GPL1001 and sample numbers GSM16985–GSM1988.

## Abbreviations

3-AT: 3-aminotriazole
DTT: dithiothreitol
ER: endoplasmic reticulum
ORF: open reading frame
S-UPR: Super-UPR
Tm: tunicamycin
UAS: upstream activating sequence
UPR: unfolded protein response
UPRE: unfolded protein response element
WT: wild-type.
