# Supplementary material for: Gcn4p and Novel Upstream Activating Sequences Regulate Targets of the Unfolded Protein Response
Source: PLoS Biol. 2004 Aug 17;2(8):e246. doi: 10.1371/journal.pbio.0020246 (PMC509306; doi:10.1371/journal.pbio.0020246)
Supplement: Table S4 — This table contains the sequences of primers and olignonucleotide sequences used in construction of plasmids for this study, as well as oligonucleotide sequences used for probes in the gel-shift analysis. (38 KB DOC). [file pbio.0020246.st004.doc]

***UPRE reporter plasmids:***

Source sequence contexts:

| Motif | ORF | Gene | Sequence (5’ context, motif, 3’ context) |
| --- | --- | --- | --- |
| 1 | YML130C | ERO1 | atacggag, tacgtg, tcataaaa |
| 2 | YJR089W | *BIR1* | ccttctgt, tagtgc, agagtctc |
| 3 | YGL222C | EDC1 | ccgggagc, ggttctgg, cgtgttta |
| 4 | YHR044C | *DOG1* | ttcttaac, gacgcgca, aagaaaaa |
| 5 | YKR050W | *TRK2* | gcctccag, cacaat, ttcacaac |
| 6 | YKL073W | *LHS1* | gctaatta, ggcgcgcg, cctcaaat |
| 7 | YJR073C | *OPI3* | caggtctt, ccacgtgg, aactgcca |
| 8 | YDL160C | *DHH1* | taagaaaa, aggacaac, acaatctt |

Insert oligo sequences:

“WB”=Watson strand, with BglII overhang

“CX”=Crick strand, with XhoI overhang.

Restriction site linkers are capitalized.

| Motif | Oligo | Sequence |
| --- | --- | --- |
| 1 | M1-WB | GATCcggagtacgtgtcatcggagtacgtgtcatcggagtacgtgtcat |
|  | M1-CX | TCGAatgacacgtactccgatgacacgtactccgatgacacgtactccg |
| 2 | M2-WB | GATCtctgttagtgcagagtctgttagtgcagagtctgttagtgcagag |
|  | M2-CX | TCGActctgcactaacagactctgcactaacagactctgcactaacaga |
| 3 | M3-WB | GATCgagcggttctggcgtgagcggttctggcgtgagcggttctggcgt |
|  | M3-CX | TCGAacgccagaaccgctcacgccagaaccgctcacgccagaaccgctc |
| 4 | M4-WB | GATCtaacgacgcgcaaagtaacgacgcgcaaagtaacgacgcgcaaag |
|  | M4-CX | TCGActttgcgcgtcgttactttgcgcgtcgttactttgcgcgtcgtta |
| 5 | M5-WB | GATCtccagcacaatttcatccagcacaatttcatccagcacaatttca |
|  | M5-CX | TCGAtgaaattgtgctggatgaaattgtgctggatgaaattgtgctgga |
| 6 | M6-WB | GATCattaggcgcgcgcctattaggcgcgcgcctattaggcgcgcgcct |
|  | M6-CX | TCGAaggcgcgcgcctaataggcgcgcgcctaataggcgcgcgcctaat |
| 7 | M7-WB | GATCtcttccacgtggaactcttccacgtggaactcttccacgtggaac |
|  | M7-CX | TCGAgttccacgtggaagagttccacgtggaagagttccacgtggaaga |
| 8 | M8-WB | GATCaaaaaggacaacacaaaaaaggacaacacaaaaaaggacaacaca |
|  | M8-CX | TCGAtgtgttgtccttttttgtgttgtccttttttgtgttgtccttttt |

## Intact promoter constructs:

PCR fragments:

| Promoter | Wildtype  construct | Mutant construct,  5’ fragment | Mutant construct,  3’ fragment |
| --- | --- | --- | --- |
| *ERO1* | oCP-184,oCP-185 | oCP-184,oCP-238 | oCP-239,oCP-185 |
| *DHH1* | oCP-186,oCP-187 | oCP-186,oCP-240 | oCP-187,oCP-241 |

Primer sequences:

Linker sequences are capitalized.

| Primer index | Description | Sequence |
| --- | --- | --- |
| oCP-184 | 5’-*BglII*-*ERO1*promoter,  5’ primer | atatAGATCTtaaattgccaaccacctacc |
| oCP-185 | 3’-*BamHI*-*ERO1*promoter,  3’ primer | gccgGGATCCtctcatgttttacctgcacg |
| oCP-186 | *DHH1*promoter-5’-*BglII* | atatAGATCTattgagccctttcaagttcc |
| oCP-187 | *ERO1*promoter-3’-*BamHI* | gccgGGATCCacccattactactattttctttc |
| oCP-238 | 5’-*ERO1*promoter-*EcoRI*,  3’ primer | gccgGAATTCtcataaaaacttgttcaatcatccttg |
| oCP-239 | 3’-*ERO1*promoter-*EcoRI*,  5’primer | gccgGAATTCctccgtatcgtttcttttttgttctcttc |
| oCP-240 | 5’-*DHH1*promoter-*NotI*,  3’primer | attaGCGGCCGCacaatcttagaactaatgggcgattg |
| oCP-241 | 3’-*DHH1*promoter-*NotI*,  5’primer | attaGCGGCCGCttttcttacgaaatttttttccctcc |

***GCN4 overexpression construct:***

The *GCN4* locus was amplified with primers bearing *EcoRI* linker and ligated into the *EcoRI* site of the 2µ-LEU2 plasmid pRS415 [Sikorski, 1989 #437]. The resulting plasmid was verified by sequencing and complementation of the slow growth phenotype of a ∆*gcn4* mutant.

Primers:

| Primer index | Description | Sequence |
| --- | --- | --- |
| oCP-321 | 5’-*EcoRI*-*GCN4* | gcgcGAATTCaatattgccgtgttccgttaccgg |
| oCP-322 | 3’-*EcoRI*-*GCN4* | gcgcGAATTCagcataatggcagttggtaagggg |

***Gel-shift probes****:*

W=Watson strand

C=Crick strand

| Probe | Strand | Sequence |
| --- | --- | --- |
| UPRE-1  (wildtype) | W | gatctcgcggcacccgaggaactggacagcgtgtcgaaaaagttgctttttttac |
|  | C | tgagtaaaaaagcaactttttcgacacgctgtccagttcctcgggtgccgcga |
| UPRE-1  (mutant) | W | gatctcgcggcacccgaggaactggacatcgtgtcgaaaaagttgctttttttac |
|  | C | tgagtaaaaaagcaactttttcgacacgatgtccagttcctcgggtgccgcga |
| UPRE-2  (wildtype) | W | aaaaaagaaacgatacggagtacgtgtcataaaaacttgttcaatcatccttgaa |
|  | C | ttcaaggattgaacaagtttctatgacacgtactccgtatcgtttcttttttt |
| UPRE-2  (mutant) | W | aaaaaagaaacgatacggagtatgtgtcataaaaacttgttcaatcatccttgaa |
|  | C | ttcaaggattgaacaagtttctatgacacatactccgtatcgtttcttttttt |
